# Comparison of diffuse correlation spectroscopy analytical models for measuring cerebral blood flow in adults

**DOI:** 10.1117/1.JBO.28.12.126005

**Published:** 2023-12-15

**Authors:** Hongting Zhao, Eashani Sathialingam, Kyle R. Cowdrick, Tara Urner, Seung Yup Lee, Shasha Bai, Feras Akbik, Owen B. Samuels, Prem Kandiah, Ofer Sadan, Erin M. Buckley

**Affiliations:** a Emory University, Georgia Institute of Technology, Wallace H. Coulter Department of Biomedical Engineering, Atlanta, Georgia, United States; b Kennesaw State University, Department of Electrical and Computer Engineering, Marietta, Georgia, United States; c Emory University, School of Medicine, Department of Pediatrics, Atlanta, Georgia, United States; d Emory University, School of Medicine, Department of Neurology and Neurosurgery, Division of Neurocritical Care, Atlanta, Georgia, United States; e Children’s Healthcare of Atlanta, Children’s Research Scholar, Atlanta, Georgia, United States

**Keywords:** diffuse correlation spectroscopy, cerebral blood flow, analytical model

## Abstract

**Significance:**

Although multilayer analytical models have been proposed to enhance brain sensitivity of diffuse correlation spectroscopy (DCS) measurements of cerebral blood flow, the traditional homogeneous model remains dominant in clinical applications. Rigorous *in vivo* comparison of these analytical models is lacking.

**Aim:**

We compare the performance of different analytical models to estimate a cerebral blood flow index (CBFi) with DCS in adults.

**Approach:**

Resting-state data were obtained on a cohort of 20 adult patients with subarachnoid hemorrhage. Data at 1 and 2.5 cm source-detector separations were analyzed with the homogenous, two-layer, and three-layer models to estimate scalp blood flow index and CBFi. The performance of each model was quantified via fitting convergence, fit stability, brain-to-scalp flow ratio (BSR), and correlation with transcranial Doppler ultrasound (TCD) measurements of cerebral blood flow velocity in the middle cerebral artery (MCA).

**Results:**

The homogeneous model has the highest pass rate (100%), lowest coefficient of variation (CV) at rest (median [IQR] at 1 Hz of 0.18 [0.13, 0.22]), and most significant correlation with MCA blood flow velocities ($R_{s}=0.59$, p=0.010) compared with both the two- and three-layer models. The multilayer model pass rate was significantly correlated with extracerebral layer thicknesses. Discarding datasets with non-physiological BSRs increased the correlation between DCS measured CBFi and TCD measured MCA velocities for all models.

**Conclusions:**

We found that the homogeneous model has the highest pass rate, lowest CV at rest, and most significant correlation with MCA blood flow velocities. Results from the multilayer models should be taken with caution because they suffer from lower pass rates and higher coefficients of variation at rest and can converge to non-physiological values for CBFi. Future work is needed to validate these models *in vivo*, and novel approaches are merited to improve the performance of the multimodel models.

## 1 Introduction

Diffuse correlation spectroscopy (DCS) is a low-cost, non-invasive optical technology for measuring blood flow. DCS measures the temporal intensity autocorrelation ($g_{2}(\tau )$) of multiply scattered light that has traveled from the source to the tissue surface. The decay rate of this curve is related to the motion of red blood cells in the underlying tissue.^1^^,^^2^ Correlation diffusion theory is used to estimate an index of blood flow (${\mathrm{cm}}^{2}/s$) from $g_{2}(\tau )$. When DCS is applied in the brain, detected photons that carry information about cerebral hemodynamics have also interacted with extracerebral layers (i.e., skull, scalp, and cerebral spinal fluid) by the nature of the non-invasive measurement. Thus, signal contamination from extracerebral layers is a challenge with DCS.^3^^,^^4^

Typically, to enhance brain sensitivity, $g_{2}(\tau )$ is measured at a single “long” source detector separation (typically 2.5 to 3 cm for continuous wave DCS) to maximize the weight of photon pathlengths that arises from the brain layer while ensuring an adequate signal-to-noise ratio (SNR). The data are then fit to the semi-infinite solution to the correlation diffusion equation (CDE) (dubbed in this work the homogenous model) to estimate an average blood flow index (BFi) of the roughly banana-shaped region that spans from the source to the detector.^5^ Although this method is the most commonly adopted due to the simplicity, the measured BFi reflects a weighted average of cerebral and extracerebral hemodynamics. Alternatively, more complex two- or three-layered models have been introduced to separate extracerebral from cerebral hemodynamics.^6^^–^^9^ These multilayered methods have shown promise both *in silico* simulations,^10^^–^^12^ in phantoms,^7^^,^^9^ and *in vivo* studies in animals,^13^ with limited application in humans.^3^^,^^4^^,^^8^^,^^14^ However, the optimal approach for DCS analysis in adults is unclear, and a rigorous comparison of these multilayer analytical methods *in vivo* is lacking.

In this study, we used a convenience sample of data collected from a cohort of 20 adult patients with subarachnoid hemorrhage (SAH)^15^ to compare the performance of the homogenous, two-layer, and three-layer models. Performance was quantified via fitting convergence, fit stability, brain-to-scalp flow ratio (BSR), and correlation with transcranial Doppler ultrasound (TCD) measurements of cerebral blood flow velocity.

## 2 Methods

### 2.1 Experimental Design

Details of the dataset used for this study can be found in our former publication.^15^ In brief, 20 adult non-traumatic SAH patients who were undergoing treatment for cerebral vasospasm in the neurocritical care unit at Emory University Hospital (Atlanta, Georgia, United States) were enrolled and included for analysis. Patients were mostly female (N=14, 70%), with a mean ± standard deviation age of 48.5±10.1 years. Patients were monitored with DCS before, during, and after administration of intrathecal nicardipine to treat cerebral vasospasm. For this study, only the baseline period of monitoring prior to drug administration was included for analysis (mean (std) duration = 2.5±0.9  min, range of 0.5 to 3 min) to isolate a steady-state period without significant physiological changes. This prospective, observational study was approved by the Emory University Institutional Review Board. Written, informed consent was obtained prior to the study initiation from all patients or their surrogates.

### 2.2 Extracerebral Layer Thickness

**Fig. 1:**
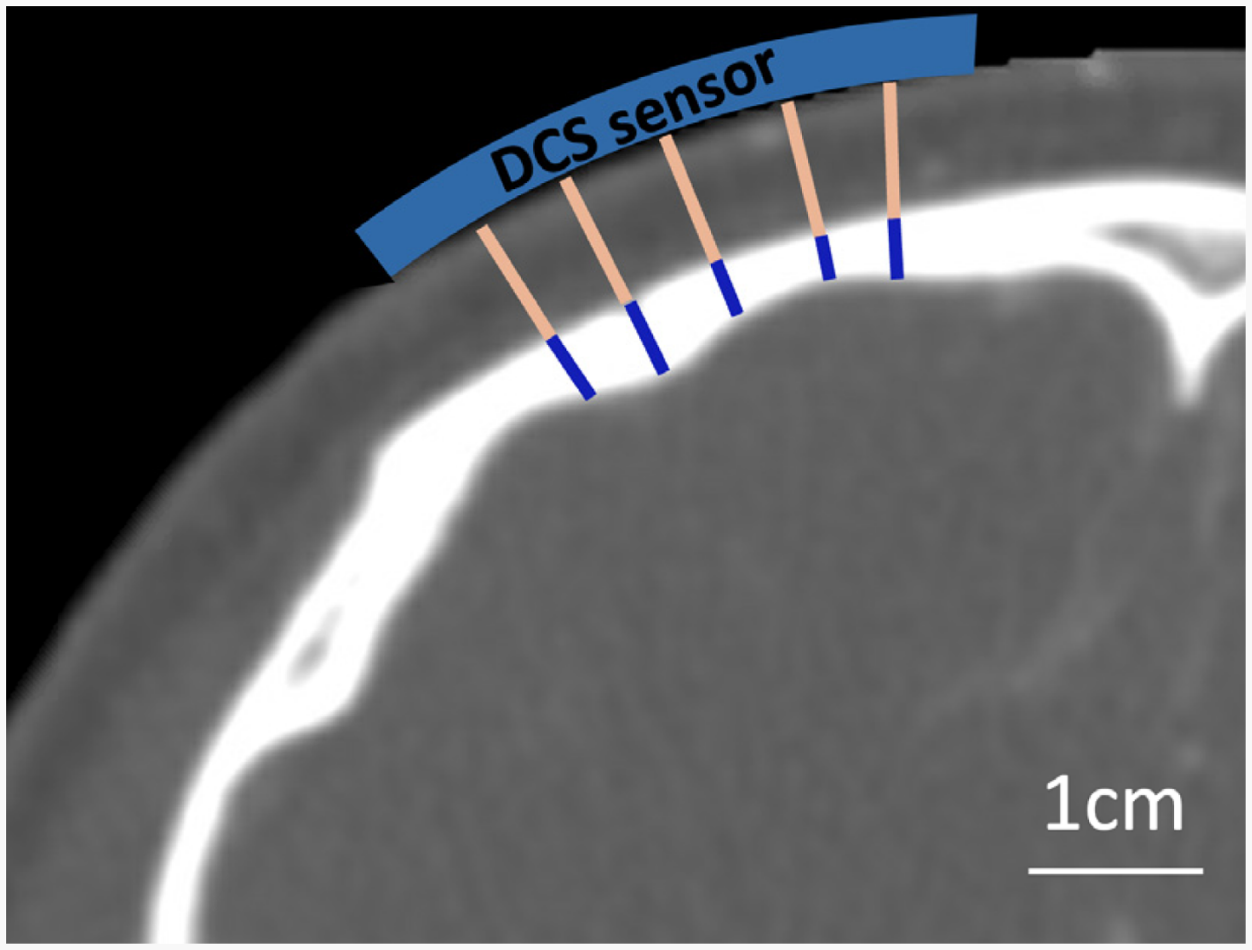
Representative measurements of scalp and skull layer thicknesses. Five separate measurements of scalp (in pink) and skull (in blue) layer thicknesses were made on an axial slice of the CT image. Measurements were made in the frontal region approximately where the DCS sensor was placed during monitoring.

All patients had head computed tomography (CT) imaging as part of standard clinical care. Axial images using a bone sequence were used to estimate skull and scalp thicknesses for DCS analysis. Five measurements of each layer thickness were made in the frontal region of the hemisphere used for DCS monitoring (Fig. 1); data were averaged to yield mean skull and scalp layer thicknesses for each patient. Patients had a mean ± standard deviation scalp thickness of 0.62±0.23  cm, skull thickness of 0.59±0.21  cm, and total depth to the brain (skull + scalp) of 1.20±0.32  cm.

### 2.3 Measurement of Macrovascular Blood Flow Velocity with TCD

TCD (Dolphin IQ, Viasonix, Tennessee, United States) was performed per usual clinical care on the morning of the study in N=18 patients. TCD data were acquired by a single operator. TCD was performed 4.5±1.9  h (mean ± standard deviation) before DCS monitoring. For comparison with DCS, the maximum of the time-averaged mean velocities for the middle cerebral artery (MCA) and anterior cerebral artery (ACA) on the hemisphere of DCS monitoring were used.^16^

### 2.4 Diffuse Correlation Spectroscopy

Data acquisition is described in depth in Sathialingam et al.^15^ In brief, DCS data were acquired at 20 Hz using an 852 nm source with one detector placed at 1.0 cm and seven detectors bundled together at 2.5 cm. The DCS sensor was place on the forehead of the hemisphere with the higher MCA velocities on TCD, suggesting worse cerebral vasospasm. If vasospasm was observed in both hemispheres, the sensor was placed on the hemisphere with higher detected light intensities to maximize the SNR. To improve the SNR, measured $g_{2}(2.5\;\mathrm{cm},\tau )$ were averaged across all seven detectors, and then $g_{2}$ at both source detector separations, ρ, were down sampled by integrating the 20 Hz data for 1, 3, or 10 s.

**Fig. 2:**
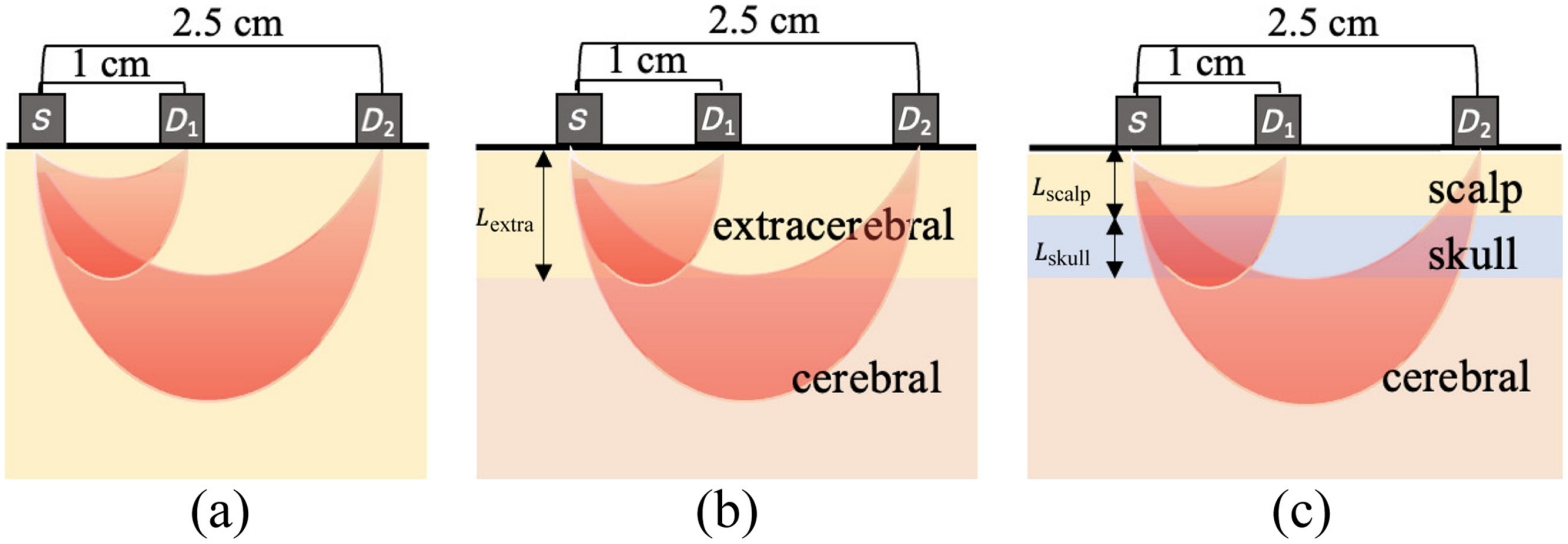
Representations of the human head. (a) Homogeneous model. (b) Two-layer model including extracerebral and cerebral layers. (c). Three-layer model including a scalp, skull, and cerebral layer. Here S denotes the source, and D denotes the detector.

To estimate brain blood flow, data were analyzed using three different models of the head (Fig. 2):

1. Homogenous model: this model treats the head as a uniform, infinitely extending medium [Fig. 2(a)]. To estimate a BFi for each ρ and time, , $g_{2}(\tau ,\rho ,t)$ were simultaneously fit for Bfi(ρ,t) and coherence factor (β(ρ,t)) using the following cost function:
  $$\chi^{2}=\overset{N_{\tau }}{\underset{k=1}{\sum }}[g_{2,\mathrm{homogeneous}}(\tau_{k},\text{BFi},\beta ,\rho ,t)-g_{2,\mathrm{measured}}(\tau_{k},\rho ,t){]}^{2},$$
  where $g_{2,\mathrm{homogeneous}}$ is the semi-infinite solution to the CDE,^1^ assuming an absorption coefficient ($\mu_{a}$) of $0.2\;{\mathrm{cm}}^{-1}$, a reduced scattering coefficient ($\mu_{s}^{\prime }$) of $10\;{\mathrm{cm}}^{-1}$, and a tissue index of refraction (n) of 1.4. $N_{\tau }$ is the number of delay times, τ; for ρ=1.0  cm, $N_{\tau }$ is the length of $g_{2}(\tau ,\rho =1.0,t)$, and for ρ=2.5  cm, $N_{\tau }$ is the number of τ for which $g_{2}(\tau ,\rho =2.5,t)>1.2$ to weight the fit toward photons that have traveled deeper.^5^ The *fminsearchbnd* function in MATLAB (Mathworks, Natick, Massachusetts, United States) was used to minimize χ with bounds for Bfi(ρ,t)∈   [1e-11, 1e-5] ${\mathrm{cm}}^{2}/s$ and β(ρ,t)∈ [0.40, 0.55]. A loose bound for Bfi was intentionally chosen to enhance the robustness of the results. Similarly, Bfi(ρ,t)and β(ρ,t) were fit simultaneously to minimize errors in the estimation of Bfi.^17^ For comparison with other models, we defined Bfi(ρ=1.0,t) as the scalp BFi (SBFi) and Bfi(ρ=2.5,t) as the cerebral BFi (CBFi).
2. Two-layer model: this model treats the head as a series of two infinitely extending slabs, in which the top slab encompasses extracerebral tissue and the bottom layer encompasses the brain [Fig. 2(b)]. To estimate the blood flow in each of these layers, $g_{2}(\tau ,\rho ,t)$ were simultaneously fit for an SBFi and CBFi using the following cost function;
  $$\chi^{2}=\overset{N_{r}}{\underset{j=1}{\sum }}\overset{N_{\tau }}{\underset{k=1}{\sum }}[g_{2,\mathrm{two}-\mathrm{layer}}(\rho_{j},\beta_{j},\tau_{k},L_{\mathrm{extra}},\mathrm{SBFi},\mathrm{CBFi})-g_{2,\mathrm{measured}}(\rho_{j},\tau_{k}){]}^{2},$$
  where $g_{2,\mathrm{two}-\mathrm{layer}}$ is the two-layer solution to the CDE,^8^ assuming $\mu_{a}$ of both layers to be $0.2\;{\mathrm{cm}}^{-1}$, $\mu_{s}^{\prime }$ of both layers to be $10\;{\mathrm{cm}}^{-1}$, the tissue index to be refraction of 1.4, and the thickness of the top layer ($L_{\mathrm{extra}}$) to be the sum of the scalp and skull thicknesses measured from the CT image (Sec. 2.3). $N_{r}$ is the number of ρ ($\rho_{j}\in \;$ [1.0, 2.5] cm), and $N_{\tau }$ is the number of τ, equal to the length of $g_{2}$. The coherence factor for each ρ ($\beta_{j}$) was assumed to be β(ρ,t) as obtained from the above homogeneous model. The *fminsearchbnd* function was used to minimize χ with bounds for   SBFi(t) and CBFi(t)∈   [1e-11, 1e-5] ${\mathrm{cm}}^{2}/s$.
3. Three-layer model: this model treats the head as a series of three infinitely extending slabs that mimic the scalp, skull, and brain layers of the head [Fig. 2I]. To estimate blood flow in each of these layers, $g_{2}(\tau ,\rho ,t)$ were simultaneously fit for a SBFi and CBFi using the following cost function:
  $$\chi^{2}=\overset{N_{r}}{\underset{j=1}{\sum }}\overset{N_{\tau }}{\underset{k=1}{\sum }}[g_{2,\mathrm{three}-\mathrm{layer}}(\rho_{j},\beta_{j},\tau_{k},L_{\mathrm{scalp}},L_{\mathrm{skull}},\mathrm{SBFi},\mathrm{CBFi})-g_{2,\mathrm{measured}}(\rho_{j},\tau_{k}){]}^{2},$$
  where $g_{2,\mathrm{three}-\mathrm{layer}}$ is the three-layer solution to the CDE,^8^ assuming $\mu_{a}$ of each layer to be $0.2\;{\mathrm{cm}}^{-1}$, $\mu_{s}^{\prime }$ of each layer to be $10\;{\mathrm{cm}}^{-1}$, a tissue index of refraction of 1.4, a negligible skull blood flow (0), and scalp and skull thicknesses ($L_{\mathrm{scalp}}$ and $L_{\mathrm{skull}}$) from CT measurements. $N_{r}$ is the number of ρ ($\rho_{j}\in$ [1.0, 2.5] cm), and $N_{\tau }$ is the number of τ, equal to the length of $g_{2}$. The coherence factor for each $\rho (\beta_{j})$ was assumed to be β(ρ,t) as obtained from the homogeneous model above. The *fminsearchbnd* function was used to minimize χ  with bounds for  SBFi(t) and CBFi(t)∈ [1e-11, 1e-5] ${\mathrm{cm}}^{2}/s$.

**Fig. 3:**
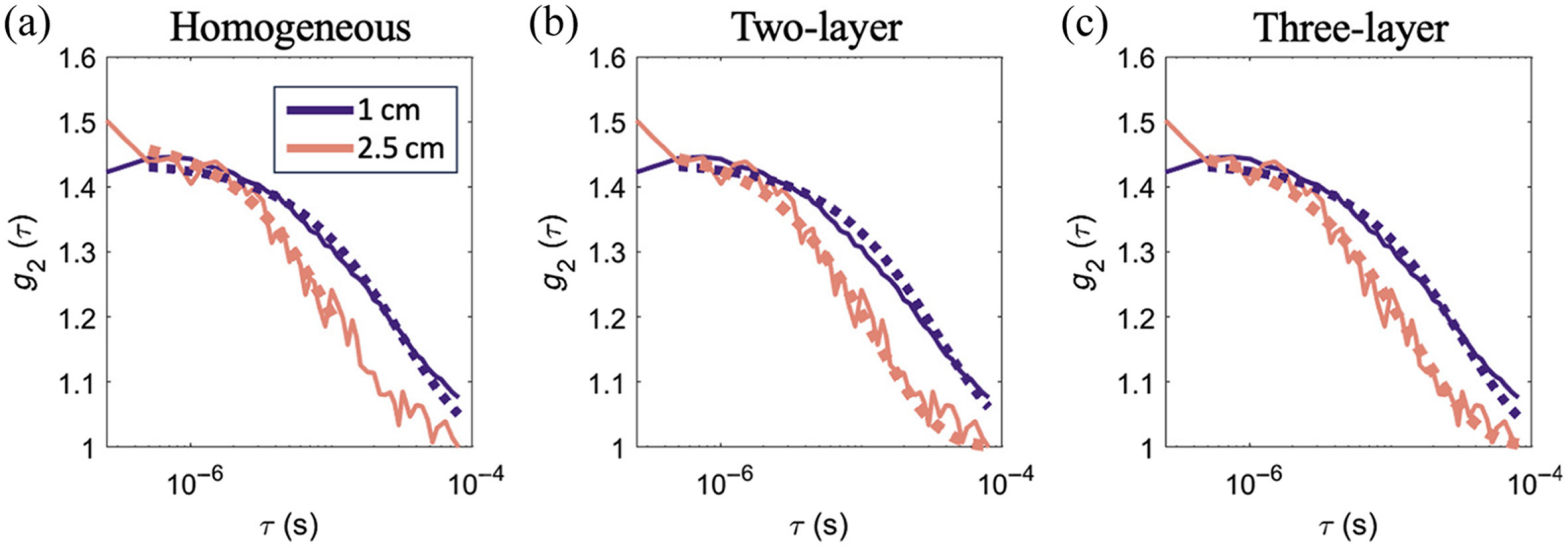
Representative intensity autocorrelation fits. Measured $g_{2}(\tau )$ at 1 (purple) and 2.5 cm (pink) fit to the (a) homogeneous, (b) two-layer, and (c) three-layer models. Solid lines denote measured data (3 s integration), and dotted lines denote the best fit. Data were obtained on a patient with scalp and skull thicknesses of 0.56 and 0.85 cm, respectively. The homogeneous model resulted in SBFi = 1.61e-8 ${\mathrm{cm}}^{2}/s$ and CBFi = 1.55e-8 ${\mathrm{cm}}^{2}/s$; the two-layer model resulted in SBFi = 1.52e-8 ${\mathrm{cm}}^{2}/s$ and CBFi = 1e-11 ${\mathrm{cm}}^{2}/s$; and the three-layer model resulted in SBFi = 0.19e-8 cm^2^/s and CBFi = 22.7e-8 ${\mathrm{cm}}^{2}/s$. Although all models generally fit the data well, the two-layer model yielded a CBFi value that fell on the fitting bounds (1e-11 ${\mathrm{cm}}^{2}/s$).

Several quality metrics were implemented for these fits. First, for each t, the fit must converge, i.e., the “exitflag” variable in *fminsearchbnd* must equal 1. Second, because we often observed fits that converged to the fitting bounds (Fig. 3), we discarded frames in which SBFi or CBFi do not fall within [2e-11, 9e-6] ${\mathrm{cm}}^{2}/s$. We defined passing datasets as those in which >50% of time points from the entire monitoring session met these two-quality metrics.

### 2.5 Statistical Analysis

Data are reported as median (interquartile range [IQR]) or count (percentage). To investigate the relationship between model pass rate and extracerebral layer thickness, Wilcoxon rank sum tests were used to test for differences in scalp, skull, and extracerebral (scalp + skull) thicknesses between datasets that passed versus failed. To evaluate the stability of each model during the monitoring period, the coefficient of variation (CV) was quantified as the ratio of standard deviation to the mean of CBFi across the monitoring window (∼2.5  min). Paired Wilcoxon signed rank tests were used to assess differences in the CV in the homogenous model versus the two- and three-layered models and for CV differences in the two- versus three-layer model. BSR was defined as the ratio of mean CBFi to mean SBFi over the monitoring period. Paired Wilcoxon signed rank tests were used to test for differences in the BSR between models. The Spearman correlation coefficient, $R_{s}$, was used to investigate the correlation between the BSR and layer thickness. Finally, to compare DCS with TCD, $R_{s}$ was used to assess the correlation between mean CBFi over the monitoring period from each model and MCA and ACA blood flow velocities. All statistical analyses were performed in MATLAB. Significance was assessed at the 0.05 level.

## 3 Results

Twenty datasets were included for analysis. The median [IQR] signal intensity was 437 (351, 478) kcps at 1 cm and 19 (17, 38) kcps at 2.5 cm. Median [IQR] β(ρ=1  cm) estimated with the homogenous model was 0.46 (0.43, 0.47) and β(ρ=2.5  cm) was 0.46 [0.44, 0.47]. No significant difference in the coherence factor was observed between source detector separations (p>0.05).

### 3.1 Fitting Quality

**Fig. 4:**
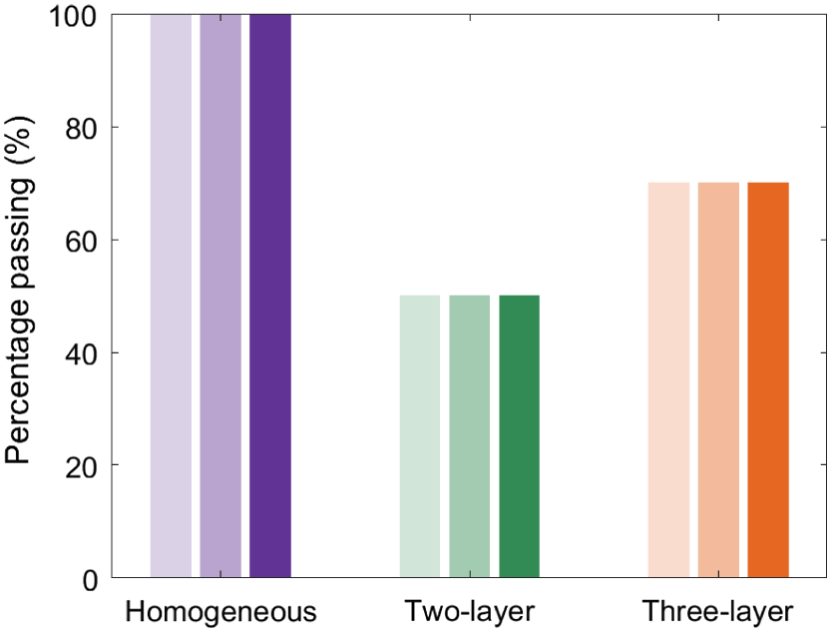
Pass rate by model. Bar plot of the percentage of datasets (N=20) that passed quality criteria when using the homogeneous (purple), two-layer (green), and three-layer models (orange). Shaded bars represent increasing SNR by averaging data 1 (light), 3 (medium), or 10 s (dark).

All datasets (100%) analyzed with the homogenous model passed the quality criteria outlined in Sec. 2.4. Median [IQR] resting-state SBFi was 0.84 [0.47, 1.10] e-8 ${\mathrm{cm}}^{2}/s$ and CBFi was 0.92 [0.60, 1.33] e-8 ${\mathrm{cm}}^{2}/s$ with this model. By contrast, 10/20 (50%) datasets passed with the two-layer model, and 14/20 (70%) datasets passed with the three-layer model (Fig. 4). For the three integration times tested (1, 3, and 10 s), the fraction of data that passed for any given model was independent of SNR (Fig. 4). For the two-layer model, median [IQR] SBFi for passing datasets was 0.58 [0.42, 1.02] e-8 ${\mathrm{cm}}^{2}/s$ and CBFi was 4.14 [2.75, 8.42] e-8 ${\mathrm{cm}}^{2}/s$. For the three-layer model, median [IQR] SBFi for passing datasets was 0.85 [0.62, 1.12] e-8 ${\mathrm{cm}}^{2}/s$ and CBFi was 5.30 [3.60, 15.65] e-8 ${\mathrm{cm}}^{2}/s$.

**Fig. 5:**
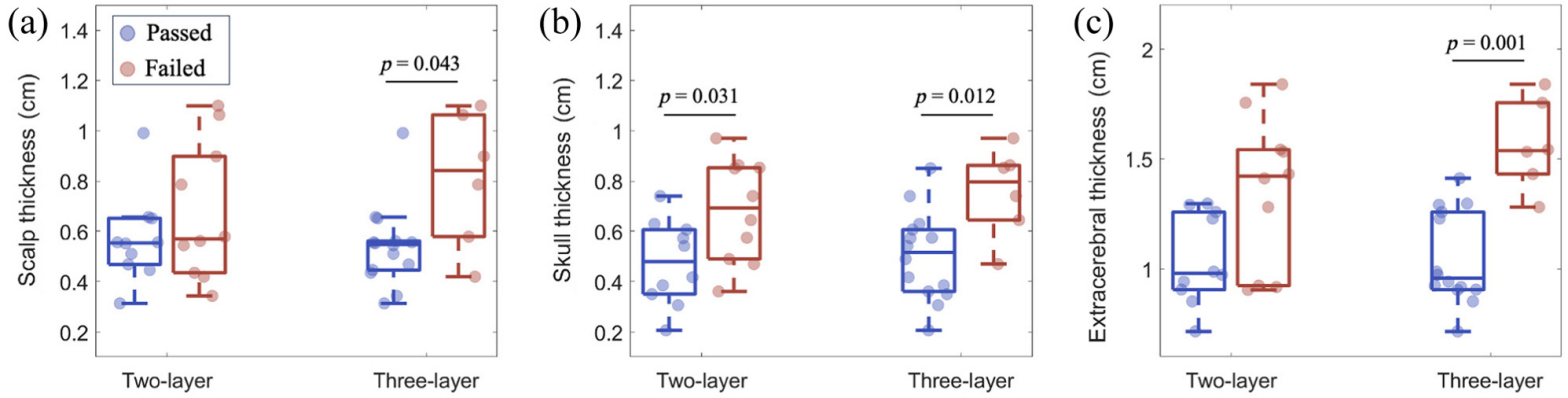
Dependence of multilayer model pass rate on the extracerebral thickness. Boxplots of (a) scalp, (b) skull, and (c) extracerebral, i.e., scalp + skull, thicknesses dichotomized by datasets that passed (blue) and failed (red) for the two- and three-layer models. p-values determined by the Wilcoxon rank sum test.

For the two- and three-layer models, the fraction of datasets that passed the quality criteria was related to the layer thickness. Both the skull and total extracerebral thicknesses were smaller in the datasets that passed versus those that failed (all p<0.05, Fig. 5). For example, the median [IQR] extracerebral thickness for passed datasets using the three-layer model was 0.96 (0.91, 1.26) cm versus 1.54 (1.43, 1. 76) cm for failed datasets (p=0.001). The scalp thickness in passed versus failed datasets also trended toward lower values although the difference was only statistically significant for the three-layer model (p=0.043).

### 3.2 Coefficient of Variation

**Fig. 6:**
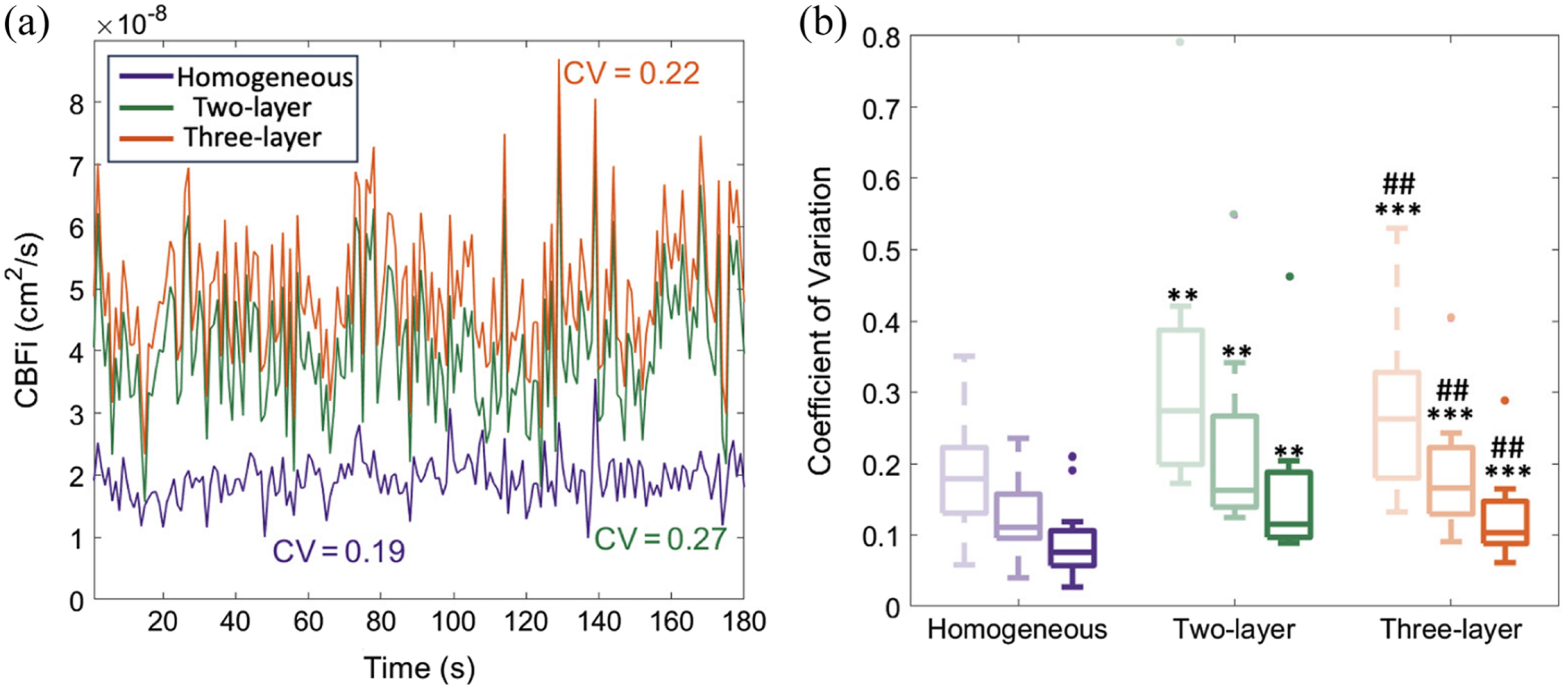
Variation in CBFi at rest. (a) Example time series of CBFi estimated with the homogeneous, two-layer, and three-layer models (in purple, green, and orange, respectively) at 1 s integration time, along with the corresponding CV. (b) Boxplots of the CV of CBFi for datasets that pass quality control with the homogeneous (purple, N=20), two-layer (green, N=10), and three-layer models (orange, N=14). Shaded boxes represent increasing SNR by averaging data 1 (light), 3 (medium), and 10 s (dark). **p<0.01 for a paired Wilcoxon signed rank test with the homogenous model. ***p<0.001 for a paired Wilcoxon signed rank test with the homogeneous model. ##p<0.01 for a paired Wilcoxon signed rank test with the two-layer model.

In general, we observed that the stability of CBFi over the resting state monitoring window was highest for the homogenous model [representative time series in Fig. 6(a)]. Figure 6(b) shows the CV of CBFi over the resting-state monitoring period for all passing datasets and all analysis models. The CV was smallest for the homogenous model. At an integration time of 1 s, the median [IQR] CV for the homogenous model was 0.18 (0.13, 0.22) versus 0.27 (0.20, 0.39) for the two-layer model and 0.26 (0.18, 0.33) for the three-layer model (both p<0.01). The CV for the two-layer model was significantly higher than that of the three-layer model for all integrations time tested (all p<0.01, N=10, paired Wilcoxon signed rank test). The CV for all analysis models decreased with increased integration times. The CV of SBFi was not statically different across models (Fig. 10).

### 3.3 Brain-to-Scalp Flow Ratio

**Fig. 7:**
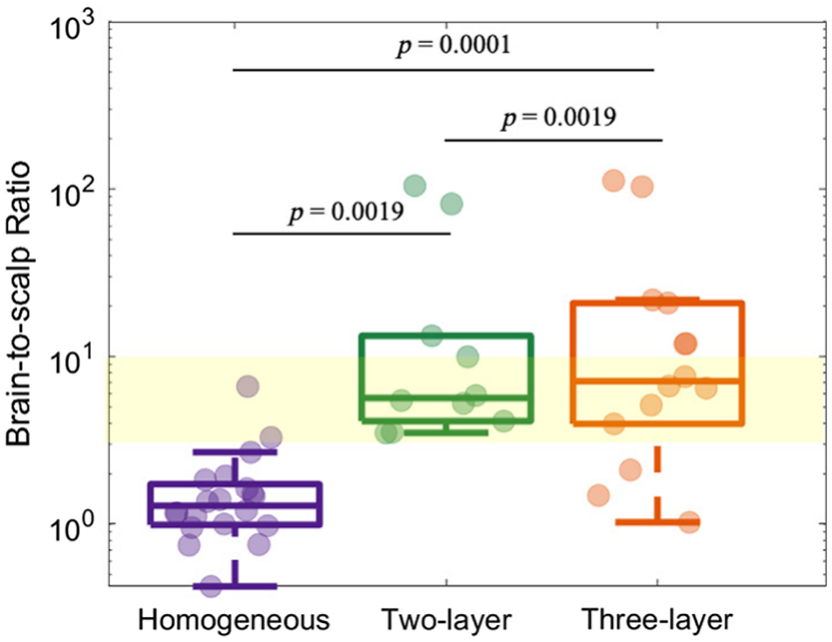
Brain-to-scalp flow ratio. Data were estimated using the homogeneous (in purple, N=20), two-layer (in green, N=10), and three-layer models (in orange, N=14). BSR within ∼3 to 10 is highlighted in the yellow region. p-values were estimated using a paired Wilcoxon signed rank test between different methods.

**Fig. 8:**
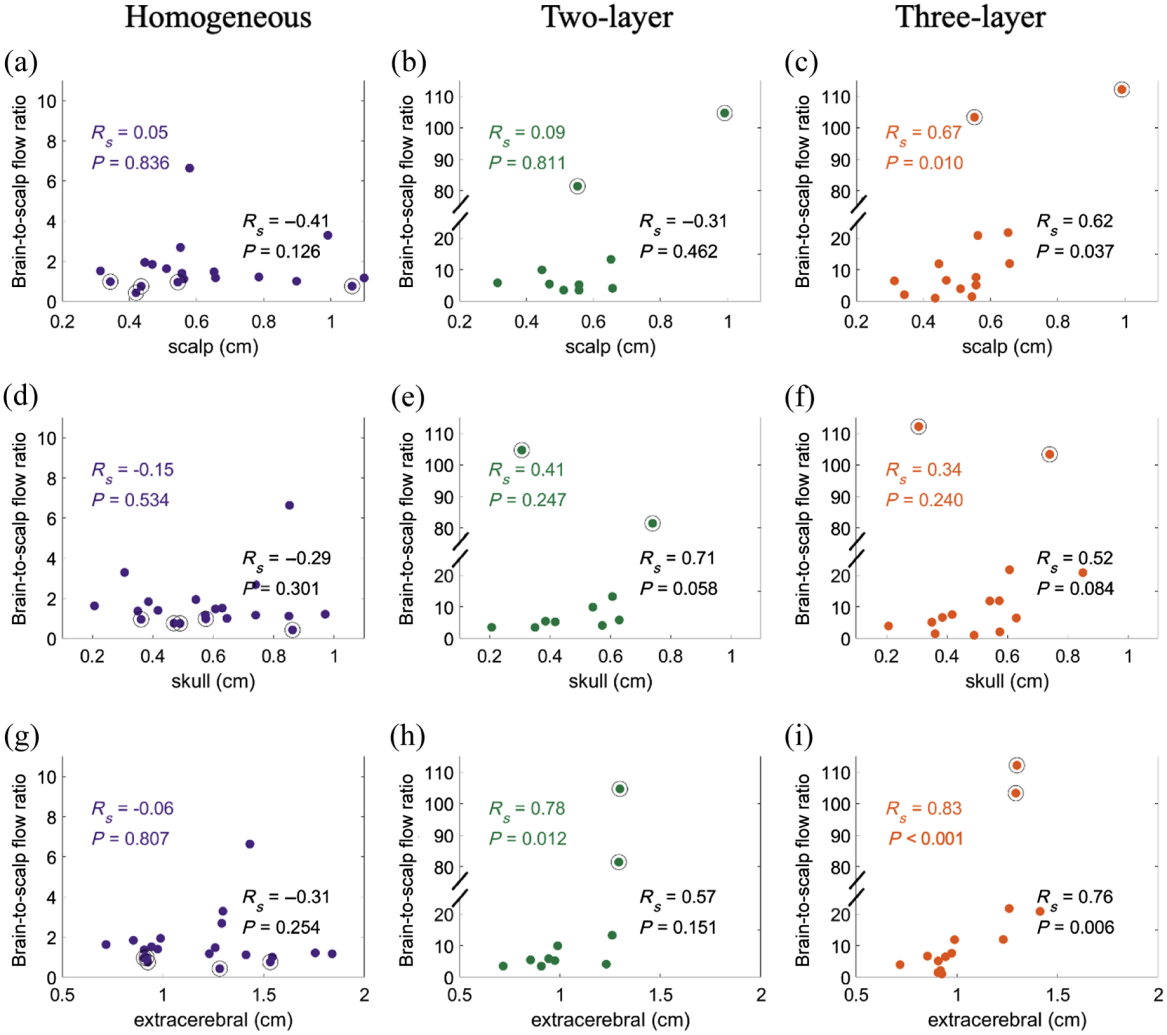
Relationship between the BSR and layer thickness. BSR as a function of (a)–(c) scalp, (d)–(f) skull, and (g)–(i) total extracerebral layer thicknesses using the homogeneous (left column, N=20), two-layer (middle column, N=10), and three-layer models (right column, N=14)). Black circles denote data points with BSR outside the range of [1, 25]. Spearman correlation coefficients and associated p-values are shown for all data (color text) and for data with BSR ∈ [1, 25] (black text).

The median BSR was highest for the three-layer model (7.4 [4.0, 21.3] versus 6.1 [5.3, 13.3] for the two-layer model and 1.3 [1.0, 1.8] for the homogeneous model, Fig. 7). For all models, the BSR was insensitive to the SNR (data not shown). Limited literature suggests that the BSR should be ∼3 to 10 for healthy adults;^18^ however, we observed several subjects with BSRs that were an order of magnitude greater than this expected range. Thus, we imposed a loose quality criterion that the BSR ∈ [1, 25]. After applying this criterion, the median [IQR] BSR was 6.6 [3.0, 11.9] for three-layer (N=12), 5.4 [3.8, 7.9] for two-layer (N=8), and 1.5 [1.2, 1.8] for homogenous (N=15). Interestingly, for the three-layer model (Fig. 8), the BSR was significantly positively correlated with the scalp (p=0.010) and extracerebral (p<0.001) thicknesses. And these trends were less pronounced when only considering the BSR ∈ [1, 25]. For the two-layer model, the BSR was only significantly positively correlated with the extracerebral thickness. But these trends were no longer significant when only considering the BSR ∈ [1, 25].

### 3.4 Correlation with TCD

**Fig. 9:**
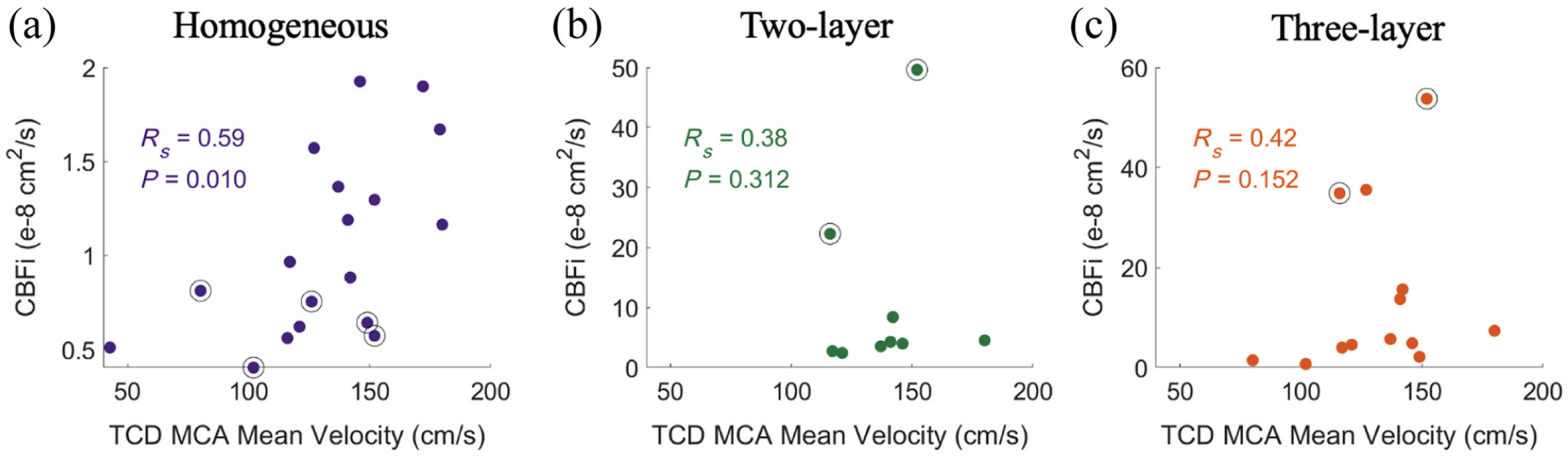
Relationship between TCD measured MCA mean velocity and DCS measured CBFi using the (a) homogeneous (N=18), (b) two-layer (N=9), and (c) three-layer models (N=13) for the datasets with both TCD data and DCS data that passed quality criteria. Black circles denote data points with BSRs outside the range of [1, 25].

A significant correlation between TCD measurements of MCA blood flow velocity and CBFi was observed with the homogeneous model ($R_{s}=0.59$, p=0.010, N=18, Fig. 9). No correlation was observed with CBFi from the two- or three-layer model. Imposing criteria that the BSR ∈ [1, 25] improved the correlation for the homogeneous model ($R_{s}=0.65$, p=0.018, N=13), and the correlation became statistically significant for the two-layer model ($R_{s}=0.79$, p=0.048, N=7) and trended toward significance for the three-layer model ($R_{s}=0.47$, p=0.146, N=11). Correlations between TCD-measurements in the ACA and CBFi from all models were not statistically significant (data not shown).

**Fig. 10:**
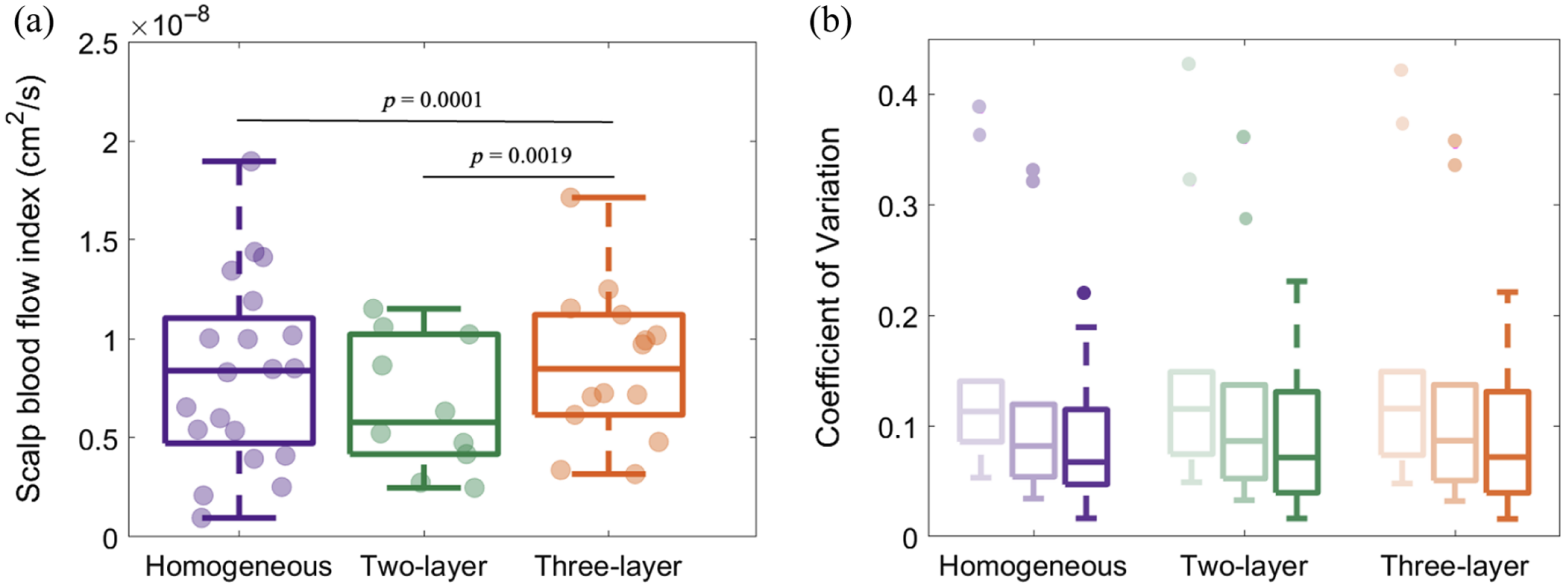
SBFi measured from all models. (a) Boxplot of SBFi for the homogeneous (in purple, N=20), two-layer (in green, N=10), and three-layer models (in orange, N=14). p-values are calculated with paired Wilcoxon signed rank test between different methods. (b) Boxplots of the CV of SBFi for 10 datasets that passed quality metrics in all models. Shaded boxes represent increasing SNR by averaging data 1 (light), 3 (medium), and 10 s (dark).

## 4 Discussion

Herein, we use a clinical dataset from a cohort of adult hemorrhagic stroke patients to test the performance of several commonly used fitting paradigms for estimating brain blood flow with DCS. Because we lacked a ground truth cerebral blood flow assessment in this dataset, we focused the investigation on fitting quality metrics as well as comparison with TCD blood flow velocities. We found that the homogeneous model has the highest pass rate, lowest CV at rest, and most significant correlation with MCA blood flow velocities. Results using the multilayer model suffer from lower pass rates and higher coefficients of variation at rest and can converge to non-physiological values for CBFi. These results highlight the challenges of implementing multilayered models in human datasets in which a large fraction of data fails to converge, which emphasizes the need for novel approaches that improve the performance of multimodel models.

The multilayered model fitting suffered from lower pass rates compared with the homogenous model (Fig. 4), which has previously been noted for the three-layer model in juvenile pigs.^13^ This trend was independent of the SNR for the integration times that we investigated; however, the pass rate will likely decrease at higher sampling frequencies in which the SNR is significantly reduced because these models have been shown to be highly sensitive to noise.^12^^,^^19^ One striking, although not surprising, finding was that the pass rate of the multilayer model fitting was significantly influenced by the extracerebral layer thickness. This trend was most prominent in the three-layer model, although it was also seen in the two-layer model. This result suggests that the multilayer models work best for subjects with thinner extracerebral layers. We speculate that this correlation is caused by limited brain sensitivity for some subjects with the 2.5 cm source detector separation used in this study. Novel approaches that improve SNR and enable larger source detector separations^20^^,^^21^ may improve the performance of these multilayer models.

In addition to fitting pass rates, we also investigated the CV of each model across the resting-state monitoring period. We found that CBFi CV was highest with the two-layer model, although both multilayered models had a significantly higher CV than the homogeneous model. Although blood flow in the brain at the sampling frequencies chosen here (1, 0.3, and 0.1 Hz) can change with respiration, neural activity, and other physiological variations, the increased CV of CBFi may indicate instability of the multilayered model fitting given the magnitude of the variations observed. For example, with TCD, it has been shown that the CV of mean cerebral blood flow velocity was 0.093,^22^ which is significantly less than the ∼0.25  CV that we observed for both multilayer models.

The BSR was significantly higher when using multilayer models compared with homogeneous model, similar to observations from Verdecchia et al.^13^ using the three-layer model in juvenile pigs. Although literature about the expected BSR is sparse, a range of 3 to 10 has been suggested as a reasonable approximation for healthy adults.^18^ We had several datasets from both the homogenous model and the multilayer models that fell well outside of this expected range. For the homogenous model, data were always discarded because of a low BSR (<1), presumably due to limited brain sensitivity. For the multilayer models, data were discarded due to a non-physiologically high BSR (>25). The reason for these large outliers is less clear; they could be due to errors in the layer thickness or assumed optical properties or to head curvature.^10^^–^^12^ Regardless, it appears that there may be crosstalk between the extracerebral layer thickness and BSR for the three-layer model as well as, to a lesser extent, the two-layer model.^19^

**Fig. 11:**
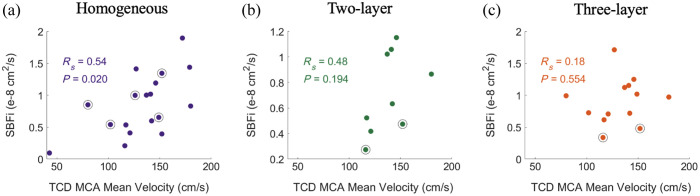
Relationship between TCD measured MCA mean velocity and DCS measured SBFi using the (a) homogeneous (N=18), (b) two-layer (N=9), and (c) three-layer models (N=13) for the datasets with both TCD data and DCS data that passed quality criteria. Black circles denote data points with BSRs outside the range of [1, 25].

We found that CBFi estimated with the homogenous model was significantly positively correlated with TCD blood flow velocities in the MCA obtained on the same day. Given that the TCD and DCS measurements were often acquired hours apart, a lack of correlation between DCS and TCD would be difficult to interpret given the patients’ possible hemodynamic instability. However, the presence of correlation is promising. We speculate that, if the measurements were taken simultaneously, the strength of the correlation between the homogenous model CBFi and MCA velocities would improve. Interestingly, a similar correlation was also observed between MCA velocities and SBFi from the homogenous model (Fig. 11), presumably due to the strong correlation between CBFi and SBFi with this model (R=0.69, p=0.001, data not shown). Similar correlations between CBFi and TCD were not seen with the multilayer models. This lack of correlation was somewhat expected given prior *in silico* work that demonstrates that these models are highly sensitive to inaccuracies in the layer thickness and brain optical properties, as well as the head curvature.^10^^–^^12^ Nevertheless, it is encouraging that, by excluding obvious outliers in BSR, a positive correlation with TCD begins to emerge for both the two- and three-layer models ($R_{s}=0.79$, p=0.048 and $R_{s}=0.47$, p=0.146, respectively). This result aligns with Wu et al.’s suggestion that the BSR could be used as a quality metric for all models to indicate whether CBFi truly reflects a marker of cerebral perfusion.^23^ Because not all data passed all three models, different sample sizes were used for the correlation analysis across the models. We acknowledge that this sample size disparity is a limitation because the statistical power to detect a significant correlation may be different due to the varying sample sizes. However, we conducted a sensitivity analysis of the nine subjects whose data passed all three models, and the results were similar to that presented in Fig. 9 ($R_{s}=0.57$, p=0.121; $R_{s}=0.38$, p=0.313; $R_{s}=0.28$, p=0.463 for the homogenous, two-layer, and three-layer models, respectively). Finally, we point out that hematocrit varied across the cohort (median [IQR] of 34 [27, 36] %). Because hematocrit can significantly confound DCS measurements,^24^ the correlation between DCS and TCD may further improve with the development of validated approaches to correct DCS for hematocrit.

Given the relatively poorer performance of the multilayer model fitting in terms of pass rates and CV, we investigated ways to improve the fitting stability of these models by adding constraints on SBFi. We tried both fixing SBFi to ${\mathrm{BFi}}_{\mathrm{homogenoeus}}(\rho =1\;\mathrm{cm})$ and constraining SBFi to ±20% of ${\mathrm{BFi}}_{\mathrm{homogenoeus}}(\rho =1\;\mathrm{cm})$, motivated by Forti et al.^19^ In general, neither of these constraints improved the multilayer model performance in terms of the pass rate or CV. We did note that fixing SBFi significantly increased the BSR with the three-layer model (7.4 [4.0, 21.3] for fitted SBFi versus 13.3 [6.2, 39.6] for fixed SBFi, p=0.002). This effect is likely because ${\mathrm{BFi}}_{\mathrm{homogeneous}}(\rho =1\;\mathrm{cm})$ does not solely reflect the scalp flow. Rather, it is likely influenced by both the scalp and skull, thereby artificially decreasing the estimate of SBFi (Fig. 10).

The main limitation of this work is that the data were acquired at a single combination of SDS (1 and 2.5 cm). Although these separations are commonly used in the continuous wave DCS literature, it remains to be seen whether these results would hold true for different SDS combinations. With improvements in DCS hardware that enable larger SDS,^25^^–^^27^ the utility of multilayered modeling should be re-evaluated, as the enhanced depth penetration of the second separation would likely improve model performance. Another limitation of this study is that it was not designed to determine which model is most accurate for use in human datasets. Future prospective validation studies against other perfusion modalities are warranted. Moreover, this study only compared analytical models during the resting state. The performance of different analytical models should be further assessed during perturbations that induce disparate scalp and brain blood flow responses.

In the future, the performance of the multilayer models and the strength of their correlations with TCD could likely be improved by implementing novel approaches that incorporate measurements of layer optical properties,^10^ as in a recent work that combines two-layer diffuse optics spectroscopy with DCS.^9^ Furthermore, more work is needed to assess the accuracy of “relative changes” in CBFi estimated with the multilayer models, which should be significantly less sensitive to these confounding variables.^11^

## 5 Conclusion

In this study, we compared the fitting quality of the homogeneous model against multilayer models using a DCS dataset collected on SAH patients. We demonstrated that the homogeneous model has the highest passing rate, lowest CV at rest, and highest correlation with TCD MCA blood flow velocity measurements. Additionally, the three-layer model outperformed the two-layer model in terms of a data pass rate. Notably, this pass rate was significantly correlated with extracerebral layer thicknesses.

## 6 Appendix: Measured SBFi from All Models

Figure 10(a) shows the SBFi for each analytical model. SBFi measured with the three-layer model was significantly higher than the homogeneous model (p=0.0001, N=14) and two-layer model (p=0.0019, N=10). No differences were observed in SBFi between the homogeneous and two-layer models (p>0.05, N=10). In addition, the CV in SBFi was not different across models [Fig. 10(b)].

A significant correlation was observed between SBFi and CBFi when using the homogeneous model ($R_{s}=0.69$, p=0.001, N=20). No correlation was observed between SBFi and CBFi when using multilayer models (both p>0.5). Consequently, a significant correlation between TCD measurements of the MCA blood flow velocity and SBFi was observed with the homogeneous model (R=0.54, p=0.020, N=18, Fig. 11). No correlation was observed between the MCA blood flow velocity and SBFi from the two- or three-layer models. These trends persist even after imposing criteria that the BSR ∈ [1, 25] ($R_{s}=0.61$, p=0.030, N=13 for the homogenous model; $R_{s}=0.57$, p=0.200, N=7 for the two-layer model; and $R_{s}=0.31$, p=0.356, N=11, for the three-layer model).

## Data Availability

Data underlying the results presented in this paper are not publicly available at this time but are available from the corresponding author upon reasonable request.
